# Influence of Sulfur for Oxygen Substitution in the Solvolytic Reactions of Chloroformate Esters and Related Compounds

**DOI:** 10.3390/ijms151018310

**Published:** 2014-10-10

**Authors:** Malcolm J. D’Souza, Dennis N. Kevill

**Affiliations:** 1 Department of Chemistry, Wesley College, 120 N. State Street, Dover, DE 19901-3875, USA; 2 Department of Chemistry and Biochemistry, Northern Illinois University, DeKalb, IL 60115-2862, USA

**Keywords:** chloroformate, chlorothioformate, chlorothionoformate, chlorodithioformate, carbamoyl chloride, thiocarbamoyl chloride, Grunwald-Winstein equation, addition-elimination, ionization

## Abstract

The replacement of oxygen within a chloroformate ester (ROCOCl) by sulfur can lead to a chlorothioformate (RSCOCl), a chlorothionoformate (ROCSCl), or a chlorodithioformate (RSCSCl). Phenyl chloroformate (PhOCOCl) reacts over the full range of solvents usually included in Grunwald-Winstein equation studies of solvolysis by an addition-elimination (A-E) pathway. At the other extreme, phenyl chlorodithioformate (PhSCSCl) reacts across the range by an ionization pathway. The phenyl chlorothioformate (PhSCOCl) and phenyl chlorothionoformate (PhOCSCl) react at remarkably similar rates in a given solvent and there is a dichotomy of behavior with the A-E pathway favored in solvents such as ethanol-water and the ionization mechanism favored in aqueous solvents rich in fluoroalcohol. Alkyl esters behave similarly but with increased tendency to ionization as the alkyl group goes from 1° to 2° to 3°. *N*,*N*-Disubstituted carbamoyl halides favor the ionization pathway as do also the considerably faster reacting thiocarbamoyl chlorides. The tendency towards ionization increases as, within the three contributing structures of the resonance hybrid for the formed cation, the atoms carrying positive charge (other than the central carbon) change from oxygen to sulfur to nitrogen, consistent with the relative stabilities of species with positive charge on these atoms.

## 1. Introduction

Compounds of the type RO(C=O)Cl can be considered as derived from formate esters by replacement of the carbonyl-attached hydrogen by chlorine, leading to the naming as chloroformates, or as derived from half-esters of carbonic acid by replacing the hydroxyl group by chlorine, leading to naming as chlorocarbonates [1]. In the older literature, both of these systems of nomenclature can be found but current usage strongly favors the naming as chloroformates. An alternative approach to naming involves a consideration of replacing one of the chlorines of carbonyl chloride (phosgene, COCl_2_) with an alkoxy or aroxy group, to give alkoxycarbonyl or aroxycarbonyl chloride. This system of naming is the most convenient to use when these compounds are being used as reagents for the introduction of protecting groups (substituents) during peptide synthesis [2,3,4]. This application is extremely important and several standard abbreviations, such as Z for benyloxycarbonyl, Boc for *tert*-butoxycarbonyl, and Fmoc for 9-fluorenylmethoxycarbonyl are employed. The importance of this derivatization during peptide synthesis is illustrated by no less than 110 entries starting with “Z”, 658 entries starting with “Boc”, and 279 entries starting with “Fmoc” in the Sigma-Aldrich 2012–2014 *Handbook of Fine Chemicals*.

These groups are just three of many groupings of the ROCO-type, which are introduced, and later removed, during peptide synthesis. Chloroformates are also used in other polymer construction applications [5] and in the development of prodrugs [6].

Other halogens can replace the chlorine at the carbonyl carbon to give a family of haloformates. However, the bromoformates and iodoformates, although examples are known [1,7,8], have found few applications. Fluoroformates have been appreciably studied [1,9] and they can be used where the chloroformate ester is insufficiently stable for subsequent use in derivatization or other synthetic procedures. In particular, simple *tert*-alkyl chloroformates are of very low stability [1,10,11] but the 1-adamantyl and *tert*-butyl fluoroformates [12] are of considerably increased stability and they have found uses in both synthetic [13,14] and mechanistic studies [15,16].

**Scheme 1:**
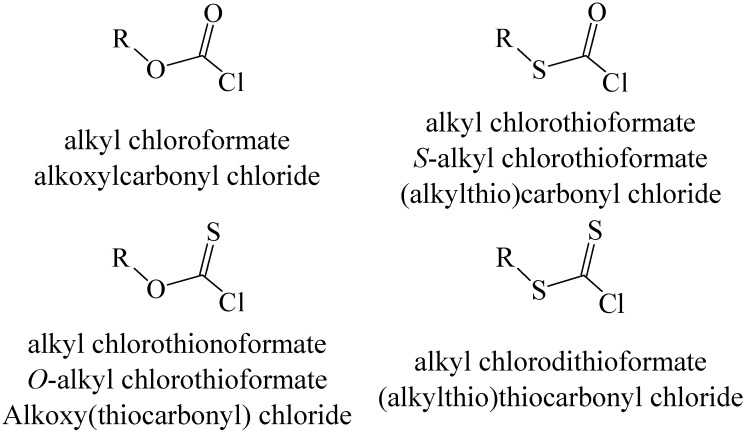
Structures and naming for sulfur-substituted alkyl chloroformates.

Another way of modifying chloroformates (or fluoroformates) is through the replacement of oxygen by sulfur. With two non-equivalent oxygens available, three types of compound can result, as indicated, with the commonly used naming (assuming an alkyl ester), in Scheme 1.

An alkoxycarbonyl group, such as Boc, is usually subsequently removed (deprotection) by hydrolysis but the benzyloxycarbonyl group (Z) can be removed by a catalyzed hydrogenolysis, usually with palladium as the catalyst [2]. Phenyl chlorothionoformate reacts with hydroxyl groups to give thiocarbonate esters, which can be reduced to the corresponding alkane derivative by use of the commercially available tributyltin hydride. This makes it, and similar chlorothionoformates, useful as reagents for initiating a deoxygenation. For example, the procedure can be used to convert ribonucleosides to deoxyribonucleosides [17,18]. For the mono-substituted derivatives, the use of “thiono” can be avoided by the use of *S*- and *O*- to indicate the identity of the atom to which the R-substituent is attached in each of the two possible isomers. In this review, we will normally use the first of the names listed for each of the structures of Scheme 1.

In addition to the derivatives of a chloroformate ester (RO(C=O)Cl) already tabulated, we can consider the replacement of the oxygen of the alkoxy group by an NR' group to give a *N*,*N*-disubstituted carbamoyl chloride, alternative naming as (dialkylamino)carbonyl chloride, and further, the influence of then replacing the remaining oxygen atom by a sulfur atom [19].

In the present review, we will concentrate upon the effect on reactivity of replacing one or more oxygen atoms by sulfur atoms within the substrate molecule. A useful technique is to consider the effect of varying the solvent composition on the specific rates (first-order rate coefficients) of a solvolysis reaction [20,21,22].

Reactions with a pathway involving rate-determining ionization will be highly sensitive to the ionizing power of the solvent, involving both overall polarity and specific solvation of the incipient ions. Reactions involving a rate-determining nucleophilic displacement of an anion will show a reduced sensitivity to changes in ionizing power and an increased sensitivity to the nucleophilicity of the solvent. Reactions involving a rate-determining addition at an unsaturated carbon atom, such as the carbonyl carbon of chloroformate esters, will show a high sensitivity to changes in solvent nucleophilicity and a reduced sensitivity to changes in solvent ionizing power, governed primarily by the solvation of the negative charge developing on the carbonyl oxygen.

Many solvolysis reactions show, even for what are generally believed to be ionization reactions, a low sensitivity to changes in solvent nucleophilicity accompanying a high sensitivity towards changes in solvent ionizing power. The reaction pathways can be considered either as involving nucleophilic solvation of the developing carbocation [23] or a loose transition state to a bimolecular (S_N_2) process [24]. The activated complexes for the two processes will be very similar in structure.

These effects can be quantitated using simple and extended forms of the Grunwald-Winstein equation [20,21,22]. The simple form (Equation (1)) is a linear free energy relationship

log (*k/k_o_*)_RX_ = *mY* + *c* (1)

(LFER) of the same general type as the Hammett equation [25], except there is now a consideration of the influence of changes in solvent composition for a given substrate, rather than changes in substrate structure in a fixed solvent. In Equation (1), *k* and *k*_o_ are the specific rates of solvolysis of a substrate RX in a given solvent and in an arbitrarily fixed standard solvent, *m* is a measure of the sensitivity to changes in solvent ionizing power *Y*, and *c* is a constant (residual) term. The standard substrate was initially *tert*-butyl chloride and the standard solvent is 80% ethanol-20% water (by volume at 25.0 °C).

Equation (1) holds well when an ionization of the type RX → R^+^ + X^−^ is involved but, provided a good selection of solvent types (so as to avoid multicollinearity) are employed in the study, poorly for S_N_2-type reactions. This is to be expected from the neglect of the important contribution from solvent nucleophilicity. This effect can be accommodated by addition of a second term (Equation (2)), in which the sensitivity *l* to changes in solvent

log (*k/k_o_*)_RX_ = *lN* + *mY* + *c* (2)

nucleophilicity (*N*) is given consideration. The original solvent nucleophilicity scale (*N*_OTs_) is based on the solvolyses of methyl *p*-toluenesulfonate [26]. An alternative scale (*N*_T_) based on the solvolyses of *S*-methyldibenzothiophenium ion [27], involving a neutral dibenzothiophene molecule as the leaving group, is now usually preferred. The development and uses of the Grunwald-Winstein scales of solvent nucleophilicity and solvent ionizing power have been reviewed [22,28,29].

Other techniques which can be applied towards a study of the mechanism of solvolytic reactions include the application of the Hammett equation to a series of solvolyses under uniform conditions except that a substituent is being varied in an aromatic ring situated in the vicinity of the reaction center [25]. The study of leaving-group ratios can be very useful for reactions at an acyl carbon. The *k*_OTs_/*k*_Br_ ratio has frequently been applied in mechanistic studies at saturated carbon [30] and, especially for studies at acyl carbon, the *k*_F_/*k*_Cl_ ratio [31] has been found to be very informative [32,33]. If the bond to the halogen is being broken in the rate-determining step (RDS), then very small values for the ratio are to be expected, because of the considerably stronger C–F bond. If only a change in the hybridization influences the C–F bond, then the stronger electron-withdrawal influences of the fluorine increases the electron deficiency at the carbonyl carbon, which favors the rate-determining addition of a nucleophilic solvent molecule and larger ratios, frequently above unity, are observed.

Entropies of activation tend to be more negative for bimolecular processes due to the need for a specific orientation of two (or more) species in the RDS. In contrast, in a unimolecular process, two ions are produced from one substrate molecule. This simple picture neglects the changes in solvation and hybridization which occur but, in practice, it is found that unimolecular reactions tend to have higher (more positive) entropies of activation than similar reactions proceeding by a bimolecular pathway [34].

Solvent deuterium isotope effects can be useful [35] but the low solubility of most organic substrates in 100% water frequently requires mixing of the H_2_O or D_2_O with an inert organic cosolvent. The situation can be simplified by use of MeOH and MeOD as the two solvents for comparison [36]. Addition-elimination (association-dissociation) substitution processes tend to involve a second nucleophilic molecule acting as a general-base [37] and this tends to raise the values for the *k*_MeOH_/*k*_MeOD_ ratio above the values for either conventional bimolecular or unimolecular pathways.

The above techniques have been very useful in comparisons of the solvolytic reactivity of a “parent” chloroformate ester with the reactivity of variously-substituted derivatives [9]. In the following narrative, consideration is given to each type of sulfur-containing analog of a chloroformate ester and then brief attention will be given to the replacement of the carbonyl oxygen of an *N*,*N*-disubstituted carbamoyl chloride by sulfur. A brief survey of these topics constituted a minor contribution to an extensive (29 pages) review of the reactions of thio, thiono, and dithio analogues of carboxylic esters with nucleophiles [38]. Methods of preparation of these compounds and of *N*,*N*-disubstituted thiocarbamoyl halides have been summarized [39].

## 2. Chlorothioformates

An early investigation of the influence of replacing the oxygen of the alkoxy group present within chloroformate esters by sulfur was carried out by Queen *et al*. [40]. The specific rates of hydrolysis in 100% H_2_O of a series of chlorothioformates (named as thiochloroformates in the paper [40]) were compared with corresponding values for chloroformates from an earlier study [35]. These comparisons are presented in Table 1.

**Table 1:** Relative specific rates for solvolyses of chloroformates and chlorothioformates esters in water at 4.6 °C.

R	Ph	CH_3_	C_2_H_5_	*n*-C_3_H_7_	*i*-C_3_H_7_	*t*-C_4_H_9_
RSCOCl	1.00 *^a^*	4.47	25.2	28.0	110.2	Fast
ROCOCl	19.4	0.636	0.388	0.424	0.958	*^b^*

*^a^* Specific rate of 1.060 × 10^−4^ s^−1^; *^b^* The *tert*-butyl chloroformate is unstable [10].

Queen suggested that the relative rate differences for the chlorothioformates were linked to conjugative and hyperconjugative release of electrons from the R-group into the d-orbitals of the sulfur [41]. For methyl chlorothioformate, a very thorough study at 19 temperatures, in the range of 0–20 °C, led to an entropy of activation of +6.4 ± 0.4 cal·mol^−1^·K^−1^. This value is considerably higher than the value of −19.1 cal·mol^−1^·K^−1^ for methyl chloroformate [35] and it was suggested, consistent with related reports [34], that these values represented unimolecular and bimolecular pathways, respectively.

The relative rates of Table 1, as R is varied, show for the chloroformate a modest decrease as one goes from methyl to primary alkyl structures followed by a modest increase on going to the secondary isopropyl ester. It was suggested that this involves an addition-elimination pathway changing over to a situation with a dominant ionization pathway. For the chlorothioformate esters, a steady increase was observed from methyl to primary to secondary structures, with the continuation to the tertiary alkyl structure leading to a specific rate too large to measure by the conductometric technique employed. This suggested that an ionization pathway for solvolysis is operative across this series of substrates.

**Scheme 2:**
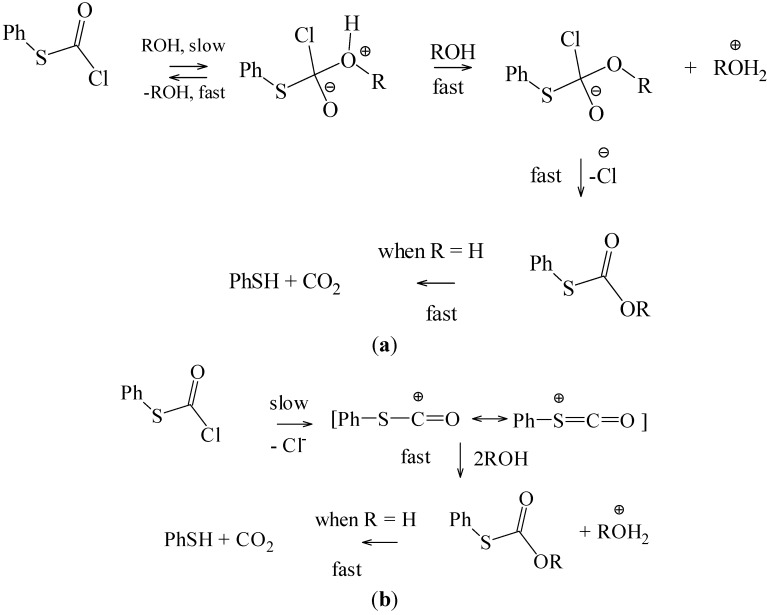
(**a**) Addition-Elimination Pathway; (**b**) Ionization Pathway.

The observation for phenyl esters, that the chlorothioformate reacted about twenty times slower than the chloroformate, (a prototype for addition-elimination reaction at an acyl carbon with the addition step rate-determining [42]), indicates that, while replacement of oxygen by sulfur enhances the S_N_1 reactions, it retards the addition-elimination pathway. The value of twenty must be regarded as a minimum value for this retardation, since it is possible that the retardation may be sufficient to lead to the observation of an appreciable ionization component for the chlorothioformate. The addition-elimination and ionization pathways, in a hydroxylic solvent (ROH), are outlined in Scheme 2.

**Figure 1:**
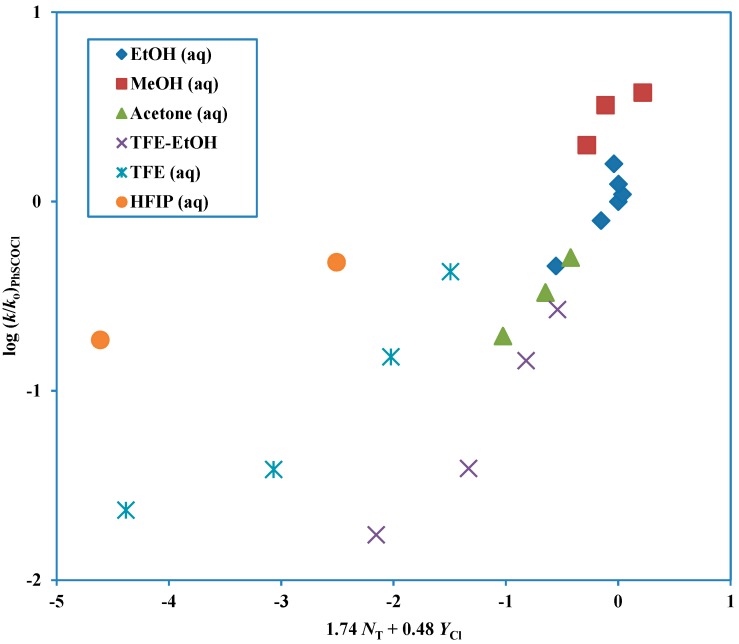
Plot of log (*k*/*k*_o_) for solvolyses of phenyl chlorothioformate at 25.0 °C against (1.74 *N*_T_ + 0.48 *Y*_Cl_). The points for the solvolyses in HFIP-H_2_O and TFE-H_2_O are not used in the correlation and they are added to the plot to show the extent of their deviation from the correlation line.

**Figure 2:**
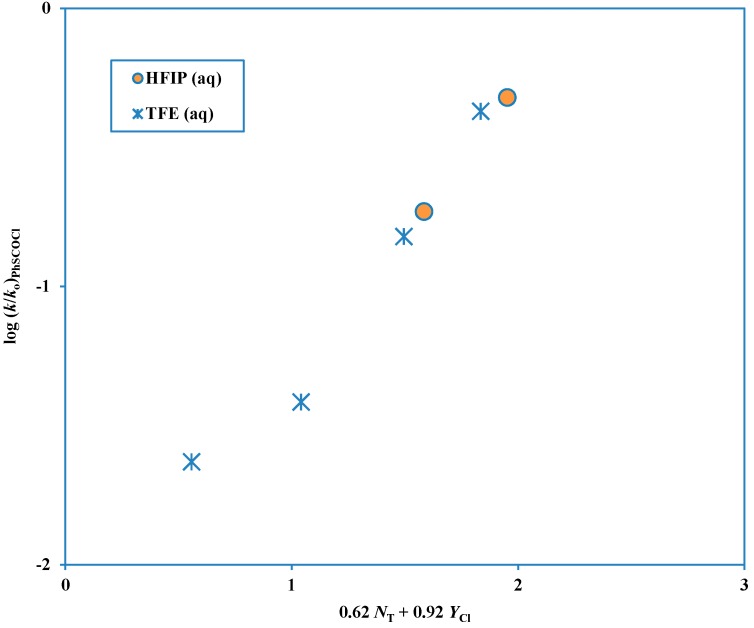
Plot of log (*k*/*k*_o_) for solvolyses in HFIP-H_2_O and TFE-H_2_O of phenyl chlorothioformate at 25.0 °C against (0.62 *N*_T_ + 0.92 *Y*_Cl_).

A study of the solvolysis of phenyl chlorothioformate as a function of solvent variation has been reported [43]. When the two-term Grunwald-Winstein equation [21,22] was applied, the data were best analyzed in terms of two plots. One plot was for solvents of high ionizing power and low nucleophilicity and the appreciable sensitivities to both solvent nucleophilicity and solvent ionizing power were consistent with an ionization mechanism with appreciable nucleophilic solvation of the developing acylium ion (a similar transition state would result from assignment as a loose S_N_2 transition state [36]). The other plot was for all other solvents and this had a higher sensitivity to changes in *N* value and a lower sensitivity to changes in *Y* value. This plot showing the deviation of the points for the solvents assigned to the alternative pathway, is shown in Figure 1 and the plot for these fluoroalcohol-containing solvents is shown in Figure 2. When there are two competing mechanisms with quite different *l* and *m* values (Equation (2)) then only solvolyses in a narrow range of solvent composition will have appreciable contributions from both mechanisms and an excellent division into two plots (as in Figure 1 and Figure 2) can be made.

For solvolyses of the phenyl chlorothioformate in 100% water, it is possible to calculate that 91% is by ionization and only 9% by addition-elimination [43], whereas for the chloroformate essentially all of the hydrolysis is by addition-elimination [42].

Expanding the study to the solvolyses of alkyl chlorothioformates, investigations have been carried out on the solvolyses of the methyl [44], ethyl [45], isobutyl [46], isopropyl [47], and *tert*-butyl [48] esters. All of these solvolyses have been carried out in a selection of solvents with widely varying solvent properties and the analyses have been in terms of the Grunwald-Winstein equations (Equations (1) and (2)). Other approaches useful for assigning mechanism, as outlined in the introduction, have also been given consideration. Values obtained using Equation (2) are presented in Table 2. Also within Table 2, for comparison purposes are values for phenyl chloroformate, ethyl chloroformate, and isopropyl chloroformate and values for *tert*-butyl chlorothioformate obtained with the use of the one-term Grunwald-Winstein equation (Equation (1)). The observation, in some instances, of a large negative value for *c* is an indication that the experimental *k*_o_ value, specific rate in 80% ethanol, relates to a faster alternative pathway.

**Table 2:** Correlation of the specific rates of solvolyses of six chlorothioformate and three chloroformate esters using the extended Grunwald-Winstein equation (Equation (2)).

Substrate	*n ^a^*	*l ^b^*	*m ^b^*	*c ^b^*	*R ^c^*	*F ^d^*	*l*/*m*
PhOCOCl *^e^*	49 *^f^*	1.66 ± 0.05	0.56 ± 0.03	0.15 ± 0.07	0.980	568	2.96 ± 0.18
PhSCOCl *^g^*	16	1.74 ± 0.17	0.48 ± 0.07	0.19 ± 0.23	0.946	55	3.63 ± 0.82
-	6	0.62 ± 0.08	0.92 ± 0.11	−2.29 ± 0.13	0.983	44	0.67 ± 0.16
MeSCOCl *^h^*	12	1.48 ± 0.18	0.44 ±0.06	0.08 ± 0.08	0.949	40	3.36 ± 0.84
-	8	0.79 ± 0.06	0.85 ± 0.07	−0.27 ± 0.18	0.987	95	0.93 ± 0.14
EtOCOCl *^e^*	28	1.56 ± 0.09	0.55 ± 0.03	0.19 ± 0.24	0.967	179	2.84 ± 0.32
-	7	0.69 ± 0.13	0.82 ± 0.16	−2.40 ± 0.27	0.946	17	0.84 ± 0.28
EtSCOCl *^i^*	19	0.66 ± 0.08	0.93 ± 0.07	−0.16 ± 0.11	0.961	96	0.71 ± 0.14
*i*-BuSCOCl *^j^*	15	0.42 ± 0.13	0.73 ± 0.09	−0.37 ± 0.13	0.961	73	0.58 ± 0.23
*i*-PrOCOCl *^e^*	9	1.35 ± 0.22	0.40 ± 0.05	0.18 ± 0.07	0.960	35	3.38 ± 0.92
-	16	0.28 ± 0.04	0.59 ± 0.04	−0.32 ± 0.06	0.982	176	0.47 ± 0.09
*i*-PrSCOCl *^k^*	19	0.38 ± 0.11	0.72 ± 0.09	−0.28 ± 0.10	0.961	97	0.53 ± 0.18
*t*-BuSCOCl *^l^*	19 *^f^*	0.13 ± 0.09	0.80 ± 0.06	−0.03 ± 0.07	0.989	365	0.16 ± 0.11
-	19 *^f^*^,*m*^	-	0.73 ± 0.03	−0.10 ± 0.05	0.988	686	-

*^a^* Number of solvents; *^b^* With accompanying standard error; *^c^* Multiple correlation coefficient; *^d^ F*-test value; *^e^* Data from [9]; *^f^* Using all available solvents; *^g^* Data from [43]; *^h^* Data from [44]; *^i^* Data from [45]; *^j^* Data from [46]; *^k^* Data from [47]; *^l^* Data from [48]; *^m^* This correlation is using Equation (1).

Phenyl chloroformate solvolyses across the complete range of solvents studied by a single mechanism of addition-elimination (A-E), with addition rate-determining [9,42]. However, although the corresponding chlorothioformate ester follows this mechanism over quite a large range of solvents, in fluoroalcohol-rich solvents an ionization mechanism is dominant. The six solvents rich in fluoroalcohol (2,2,2-trifluoroethanol, TFE and 1,1,1,3,3,3-hexafluoro-2-propanol, HFIP) deviate markedly from a plot based on the other solvents (Figure 1) and these solvents give a second plot (Figure 2) with a much lower sensitivity to solvent nucleophilicity and a much higher sensitivity to solvent ionizing power [43].

Methyl chlorothioformate solvolyzes, like the corresponding chloroformate [9,49] over a large portion of the range of solvents by the A-E mechanism. There is, however, again a wider range of the more ionizing and less nucleophilic solvents for which an ionization mechanism dominates. Very similar behavior, as regards the operation of the two reaction channels, is observed for ethyl chloroformate [9,45] and it appears that the introduction of sulfur for the methoxy-oxygen or the introduction of an α-methyl group within methyl chloroformate have, in this regard, similar influences upon the division between the two reaction channels.

The ethyl chlorothioformate solvolyzes predominantly by the ionization mechanism and only in three of the studied solvents was the addition-elimination pathway dominant: ethanol, methanol, and 90% ethanol [45]. Similarly, the primary isobutyl chlorothioformate also reacts primarily by the ionization pathway [46], with evidence for a dominant A-E pathway only in 100% and 90% ethanol, 100% and 90% methanol, and 20% TFE-80% ethanol.

**Scheme 3:**
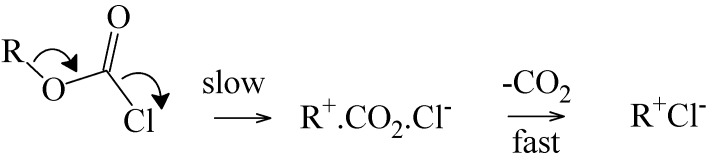
Concerted Ionization-Fragmentation Reaction of 2° and 3° Alkyl Chloroformates.

The secondary isopropyl chlorothioformate, based on the above observations coupled with the observation that isopropyl chloroformate has two fairly equally balanced regions where A-E and ionization reactions dominate [9,50,51], would be expected to have the ionization pathway dominant over a large range of solvents. Indeed, only for 100% ethanol was there an upward deviation from the correlation line, indicating for this solvent a superimposed A-E pathway [47]. It has been suggested [9,35] that a likely mechanism for solvolyses of 2° and 3° alkyl chloroformates involves a concerted fragmentation reaction (Scheme 3) leading, in the present context, to the isopropyl carbenium ion, with this then combining with the chloride ion, deprotonating to give propene plus HCl, or adding a solvent molecule.

**Scheme 4:**
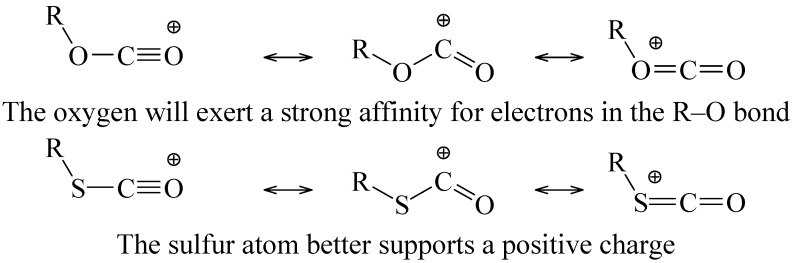
Resonance stabilized carboxylium ions from ionization of chloroformate or chlorothioformate esters.

It cannot be automatically assumed that 2° and 3° chlorothioformates will follow this pathway, especially because, although *tert*-butyl chloroformate is low stability [10], the *tert*-butyl chlorothioformate is sufficiently stable for it to be commercially available [48]. Indeed, the observation by Queen and co-workers of 2-propanethiol as the major product from the hydrolysis in pure water of isopropyl chlorothioformate [40] requires the retention of the isopropyl-sulfur bond throughout the pathway for this solvolysis. This could be a consequence of sulfur being better able to support a positive charge in the resonance-stabilized carboxylium than oxygen (Scheme 4). It is possible that a very low stability for the carboxylium ion from the chloroformate in the presence of an electron-donating alkyl group leads to an enforced concerted process for the fragmentation [52].

while the conditions are very different from solvolysis, it is of interest that methyl and ethyl chlorothioformates interact with antimony pentafluoride in liquid SO_2_ or SO_2_ClF to give alkylthiocarbonyl cations, with retention of COS, while the corresponding chloroformates lose CO_2_ to give alkyl fluoroantimonates [53] (a rapid exchange of chlorine with fluorine in the excess SbF_5_ occurs). This strongly supports the thesis that the alkylthiocarbonyl cation is more stable than the corresponding alkylcarbonyl cation. Further support comes from a calculation showing that bond dissociation energies are consistent with a simple cleavage of the C–Cl bond of a chlorothioformate being a relatively favorable process [54].

The *t*-butyl chlorothioformate shows a very low (0.13 ± 0.09) sensitivity to changes in solvent nucleophilicity coupled with a high (0.80 ± 0.06) sensitivity to changes in solvent ionizing power when the extended equation (Equation (2)) is applied. Indeed the correlation coefficient is reduced only from 0.989 to 0.988 when the original one-term Grunwald-Winstein equation (Equation (1)) is applied. As one would then predict, the *F*-test value is almost doubled on going to the one-term equation (last entry in Table 2). One can conclude that the solvolyses are best correlated by the one-term equation across the full range of solvents studied and an ionization process (Scheme 2) is operative.

The ratio of specific rates in methanol and methan (ol-*d*), *k*_MeOH_/*k*_MeOD_, was found to be 1.39 ± 0.01, very similar to the value for *t*-butyl fluoroformate of 1.26 ± 0.02 [16] and these values are within the range to be expected for a unimolecular pathway. In the trichloro-derivative of *tert*-butyl chloride, 2,2,2-trichloro-1,1-dimethylethyl chloroformate, the electron-withdrawing chlorides lead to an A-E mechanism [55,56] and an increased value of 2.14 ± 0.03 is obtained [56]. Similarly, values of 2.17 ± 0.03 have been obtained for *n*-propyl chloroformate [57] and of 2.03 ± 0.01 for isobutyl chloroformate [58] methanolyses. The higher A-E values reflect, in part, the involvement of a second methanol [or methan (ol-*d*)] molecule as a general-base [37,59].

## 3. Chlorothionoformates

In addition to their use as reagents for introducing protecting groups during peptide synthesis [3], chlorothionoformates (ROCSCl) have been found to be of use in the preparation of thiocarbonate esters, nitriles, and isonitriles [60,61] and in the dealkylation of tertiary amines [62,63]. The important reactions with amines have been the subject of several mechanistic studies [38,64,65,66,67,68]. This topic has already been reviewed twice [38,65] and we will mention just the interesting example of nucleophilic catalysis by pyridine in the hydrolysis of chlorothionoformate esters [66].

Initial mechanistic studies involved the hydrolysis in 100% H_2_O and 70% acetone [69], including a comparison with the earlier studies in 100% H_2_O of the corresponding chlorothioformate [40] and chloroformate [35]. At 4.8 °C, in 100% H_2_O, it was found that, for the phenyl esters, the chlorothionoformate reacted at almost one-third the rate of the corresponding chlorothioformate ester and the reaction was 55 times slower than that for phenyl chloroformate [69].

For the identical hydrolyses of the methyl esters, the chloroformate was now the slowest, 7 times slower than the chlorothioformate and 2.6 times slower than the chlorothionoformate. For the ethyl esters, the chlorothioformate and chlorothionoformate hydrolyzed at about the same rate, which was about sixty times higher than for the chloroformate. The results can be rationalized in terms of a dominant addition-elimination pathway for the hydrolyses of the phenyl, methyl, and ethyl chloroformates, which then acquires a superimposed ionization pathway for the isopropyl ester. For the corresponding chlorothioformate, a dominant ionization pathway for the hydrolyses appears to operate over the full range of structures (see values in Table 1). The 100% H_2_O solvolyses of the chlorothionoformate esters were only studied for phenyl, methyl, and ethyl, and over this limited range of structures, the relative rates (Ph, 1.0; Me, 4.6; Et, 68) were again consistent with a dominant ionization pathway.

More recent studies have concentrated on aryl chlorothionoformates. The extended Grunwald-Winstein equation (Equation (2)) has been applied to the solvolyses of phenyl chlorothionoformate [70,71,72], *p*-methylphenyl (tolyl) chlorothionoformate [73], *p*-chlorophenyl chlorothionoformate [73], and *p*-fluorophenyl chlorothionoformate [74]. The values obtained from these correlations are summarized in Table 3.

**Table 3:** Correlation of the specific rates of solvolyses of four aryl chlorothionoformates, phenyl fluorothionoformate and phenyl chlorodithioformate using the extended Grunwald-Winstein equation (Equation (2)).

Substrate	*n ^a^*	*l ^b^*	*m ^b^*	*c ^b^*	*R ^c^*	*F ^d^*	*l*/*m*
PhOCSCl *^e^*	9 *^f^*	1.88 ± 0.28	0.56 ± 0.15	0.38 ± 0.15	0.950	28	3.36 ± 1.36
-	18 *^g^*	0.34 ± 0.05	0.93 ± 0.09	−2.54 ± 0.34	0.955	77	0.37 ± 0.08
*p*-MeC_6_H_4_OCSCl *^h^*	13 *^i^*	1.63 ± 0.31	0.46 ± 0.10	0.30 ± 0.12	0.881	17	3.54 ± 1.42
-	7 *^j^*	0.45 ± 0.13	1.07 ± 0.14	−2.25 ± 0.20	0.986	69	0.42 ± 0.16
*p*-FC_6_H_4_OCSCl *^k^*	10 *^l^*	1.76 ± 0.28	0.54 ±0.15	0.34 ± 0.15	0.943	28	3.26 ± 1.41
-	5 *^m^*	0.53± 0.18	0.89 ± 0.18	−2.66 ± 0.35	0.967	15	0.60 ± 0.31
*p*-ClC_6_H_4_OCSCl *^h^*	13 *^i^*	1.79 ± 0.16	0.45 ± 0.07	−0.05 ± 0.09	0.966	69	3.98 ± 0.93
-	6 *^j^*	0.43 ± 0.17	0.82 ± 0.20	−3.45 ± 0.40	0.913	10	0.52 ± 0.29
PhOCSF *^n^*	22	1.32 ± 0.13	0.39 ± 0.08	−0.02 ± 0.10	0.952	95	3.38 ± 1.00
PhSCSCl *^o^*	31	0.69 ± 0.05	0.95 ± 0.03	0.18 ± 0.05	0.987	521	0.72 ± 0.07

*^a–d^* See footnotes to Table 2; *^e^* Rate data from [70] and [71] and correlation data from [72]; *^f^* In 100%–80% ethanol and methanol, 80% acetone, 80T-20E, and 60T-40E (T and E represent 2,2,2-trifluoroethanol and ethanol, respectively); *^g^* In H_2_O, 30%–10% ethanol, methanol, and acetone, and all TFE-H_2_O and HFIP-H_2_O solvents; *^h^* From [73]; *^i^* Excluding data points for TFE-H_2_O, HFIP H_2_O, and 80T-20E; *^j^* For the solvents indicated as excluded in footnote *i*; *^k^* From [74]; *^l^* Excluding data points in 97%–70% HFIP and 97%–90% TFE; *^m^* For the solvents indicated as excluded in footnote *l*; *^n^* From [75]; *^o^* From [72].

Phenyl chlorothionoformate solvolyzes in a wide variety of solvents at a rate very close to that observed for the isomeric phenyl chlorothioformate. Further, the groupings of the solvents into those with a dominant A-E mechanism and those with a dominant ionization mechanism were similar in constitution. For eleven solvents favoring the A-E pathway the *k*_PhOCSCl_/*k*_PhSCOCl_ ratio ranged from 0.36 to 0.58 and for six fluoroalcohol-water solvents the corresponding ratio ranged from 0.97 to 1.14 [70]. McKinnon and Queen [69] had previously determined a ratio of 0.35 in 100% H_2_O.

Similarly the sets of *l* and *m* values obtained in the region involving the A-E pathway are similar to each other and, also, to those for PhOCOCl solvolyses, which follow the A-E pathway over the full range of solvents (Table 2 and Table 3). Also, the values for the ionization pathway for the PhSCOCl and PhOCSCl show virtually identical sensitivities to changes in solvent ionizing power (*m* values) at 0.92 and 0.93 coupled with sensitivity towards changes in solvent nucleophilicity, somewhat greater at 0.62 for the chlorothioformate than for the chlorothionoformate, which has a value of 0.34.

For hydrolysis in 100% water, the methyl chlorothionoformate exhibits an entropy of activation of +13.5 cal·mol^−1^·K^−1^ [69], considerably higher than the value of −19.1 cal·mol^−1^·K^−1^ [35] from the corresponding temperature variation study of the hydrolyses of methyl chloroformate. Also supporting the assignments of mechanism in 100% H_2_O were the observations of solvent isotope effect (*k*_H_2_O_/*k*_D_2_O_) of 1.28 and 1.89, respectively [35,69].

Rumanian investigators carried out a study of the specific rates of hydrolysis of phenyl chlorothionoformate and eight ring substituted derivatives in 65% acetone [76]. They found for each substrate a very negative entropy of activation (−26 to −30 cal·mol^−1^·K^−1^) indicating all to be reacting by a bimolecular, presumably A-E, pathway.

A Hammett treatment using σ° values led to a ϱ value of 1.26, somewhat less than the 1.59 for the corresponding chloroformates, but still indicative of a bimolecular pathway. They found [76] similar *k*_H_2_O_/*k*_D_2_O_ solvent isotope effects in 65% acetone for chlorothionoformates and chloroformates. These studies strongly indicate an addition-elimination pathway with addition rate-determining for the solvolyses in 65% acetone for phenyl chlorothionoformate, based upon similarity in behavior to phenyl chloroformate, for which there is considerable evidence for the operation of such a mechanism.

Koo and coworkers reported [71] on the solvolyses of phenyl chlorothionoformate in methanol-water and ethanol-water over the full range of solvent composition, including the pure solvents, and also 80%–10% acetone-water. Although they had 29 data points, their overall mix of solvents was not good and in particular no solvents containing a fluoroalcohol were included in the study. Where their data coincided there was excellent agreement in the alcohol-solvents and fair agreement for two acetone-water solvents with a slightly earlier submitted report [70]. Their data was useful in a subsequent comprehensive Grunwald-Winstein equation analysis [72], discussed earlier and reported in Table 3. They obtained a *k*_MeOH_/*k*_MeOD_ value of 2.02 and a *k*_H_2_O_/*k*_D_2_O_ value of 1.45 in 10% water, values consistent with a bimolecular pathway in methanol and a predominantly ionization pathway in water.

**Figure 3:**
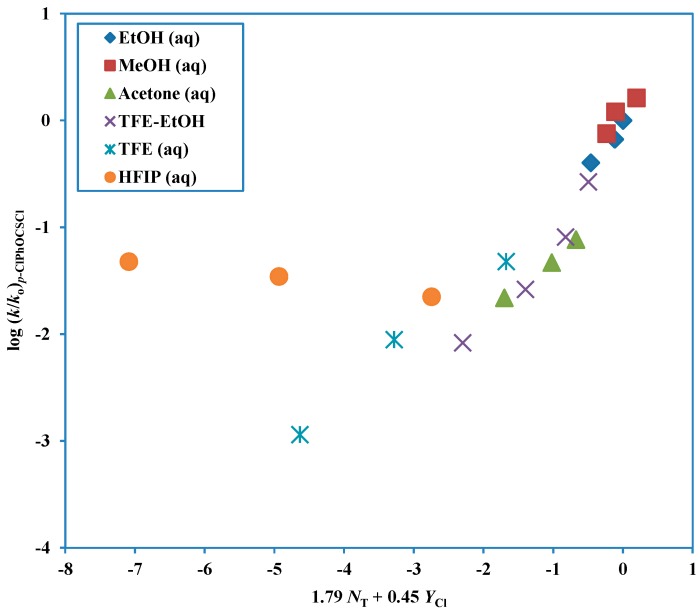
Plot of log (*k*/*k*_o_) for solvolyses of *p*-chlorophenyl chlorothionoformate against (1.79 *N*_T_ + 0.45 *Y*_Cl_) in nineteen pure and binary solvents. The points for the solvolyses in HFIP-H_2_O and TFE-H_2_O are not used in the correlation and they are added to the plot to show the extent of their deviation from the correlation line.

The previously discussed Hammett plots [76,77], for hydrolysis in 65% acetone, indicated a rate-determining nucleophilic attack. More recently, extensive Grunwald-Winstein correlations have been carried out with the electron supplying *p*-Me substituent and the electron-withdrawing *p*-Cl substituent at 25.0 °C [73]. Both of these substrates and also one with the *p*-F substituent at 35.0 °C [74] show a break in the G-W correlations which is consistent with that observed for the parent (*p*-H) compound. This behavior indicated an A-E pathway and, for the solvents rich in fluoroalcohol, an ionizing pathway (Table 3). This dichotomy can be shown, in simple terms, from relative-rates in two extreme solvents: 100% EtOH and 97% HFIP. In 100% EtOH, at 25.0 °C, the relative rates are *p*-Me, 1.0; *p*-H, 2.1; *p*-Cl, 10.5, consistent with electron-withdrawing substituent favoring a rate-determining nucleophilic attack. In 97% HFIP, the order of the relative rates is reversed: *p*-Me, 1.0; *p*-H, 0.40; *p*-Cl, 0.046, consistent with electron-withdrawal substituents hindering the ionization reaction involving expulsion of a chloride anion. The extended Grunwald-Winstein equation plot for the addition-elimination pathway is shown in Figure 3.

Another way of investigating the change in mechanism is in terms of the *l*/*m* ratios listed in Table 3. It can be seen that, for *p*-Me, *p*-H, *p*-F, and *p*-Cl substituents, the ratios are fairly constant at 3.54, 3.36, 3.26, and 3.98 for the A-E pathway and at 0.37, 0.42, 0.60, and 0.45 for the ionization pathway.

Although there have been several studies of fluoroformate esters [9], no examples of kinetic studies of fluorothioformate esters were found. There is, however, a report of a kinetic study of a fluorothionoformate ester [75]. Phenyl fluorothionoformate was prepared from commercially available chlorothionoformate as described by Bay [78].

The corresponding chlorothioformate reacted in Grunwald-Winstein equation studies (Table 3) by a dominant addition-elimination pathway in solvents of relatively high nucleophilicity and low ionizing power, which converted over to a dominant ionization pathway in aqueous fluoroalcohols. Since the strong carbon-fluorine bond severely limits the operation of an ionization mechanism, as shown by the *k*_F_/*k*_Cl_ value of 3.3 × 10^−8^ for the hydrolysis in 100% H_2_O of *p*-dimethylaminobenzoyl halides [32], the ratio is a powerful indicator of the reaction mechanism under the solvolysis conditions [31,32].

For the solvolyses of phenyl fluorothionoformate, the extended Grunwald-Winstein equation gave a linear plot over the full range of solvents with *l* and *m* values, consistent with the operation of an addition-elimination mechanism with the addition step rate-determining. The correlation data is presented in Table 3. The *l*/*m* ratio of 3.38 is within the range of values previously found for the A-E branch of the correlation analyses of aryl chlorothionoformate solvolyses.

**Figure 4:**
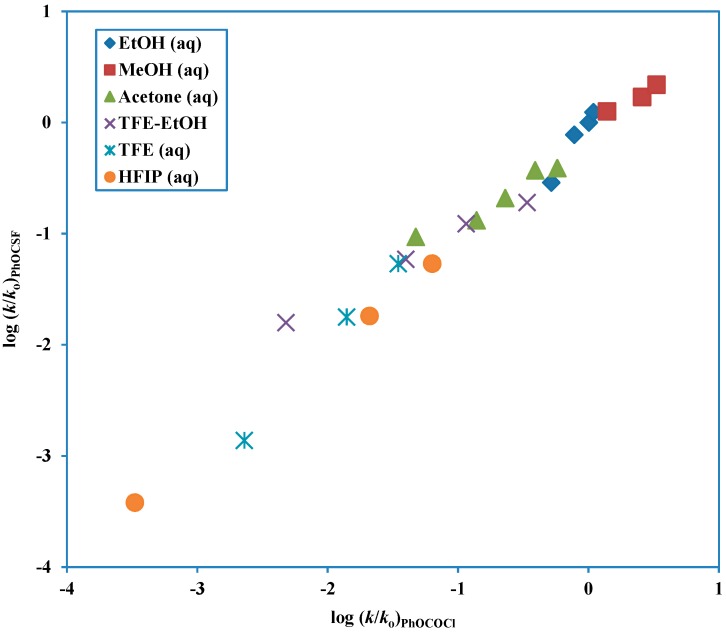
Plot of log (*k*/*k*_o_) for solvolyses of phenyl fluorothionoformate at 10.0 °C against log (*k*/*k*_o_) for solvolyses of phenyl chloroformate at 25.0 °C.

Another tool which can be used to test for mechanism is to do a direct correlation of the log (*k*/*k*_o_) values against the corresponding values for the solvolysis of a substrate with previously assigned mechanism, such in the use of phenyl chloroformate solvolyses as a prototype for a chloroformate solvolyzing by the addition-elimination mechanism. This more direct approach, using similarity models and removing the need to have scales of solvent properties available has been favored and extensively used by Bentley [79]. In comparisons of this type, a good linear correlation will be obtained if the *l*/*m* ratios for the two solvolyses are similar. A plot of this type for phenyl fluorothionoformate log (*k*/*k*_o_) values against the corresponding values for phenyl chloroformate (Figure 4) is nicely linear, consistent with *l*/*m* values of 3.38 (Table 3) and 2.96 (Table 2), respectively. The slope, actually at a value of 0.92 can be estimated as the ratio of either the *l* or the *m* values to give estimates of 0.80 and 0.70, respectively.

As one predicts from the enormous retardation, on replacing chlorine by fluorine, to be expected for the competing ionization pathway, a single Grunwald-Winstein correlation (for the A-E pathway) can be applied across the full range of the studied solvent compositions. Also, the *k*_MeOH_/*k*_MeOD_ value of 2.11 ± 0.02 is consistent with the A-E pathway. At 25.0 °C, the *k*_F_/*k*_Cl_ ratio was 493 in 100% methanol, 551 in 100% ethanol, 867 in 80% ethanol, 64 in 70% TFE, and 0.21 in 90% HFIP. The considerably lower ratios in the solvents with high fluoroalcohol content follow from the *k*_Cl_ value having a large superimposed component from a dominant ionization pathway.

## 4. Chlorodithioformates

The preparation and synthetic applications of chlorodithioformate esters have been reviewed [80]. They have been used to provide hydrophobic prodrugs by interaction with nucleic acid bases, nucleosides, and nucleotides [81].

McKinnon and Queen [69] found that, relative to the chlorothioformates and chlorothionoformates, the chlorodithioformates were of very low solubility in water and, accordingly, they were studied in 70% acetone. Temperature variation for the solvolyses of the methyl ester led to an entropy of activation of −3.7 cal·mol^−1^·K^−1^, consistent with an ionization pathway. An ionization pathway was also indicated by the relative rates for a series of chlorodithioformate esters in 70% acetone at 4.9 °C: C_6_H_5_ (1.0); CH_3_ (5.1); C_2_H_5_ (32); *i*-C_3_H_7_ (170).

**Scheme 5:**
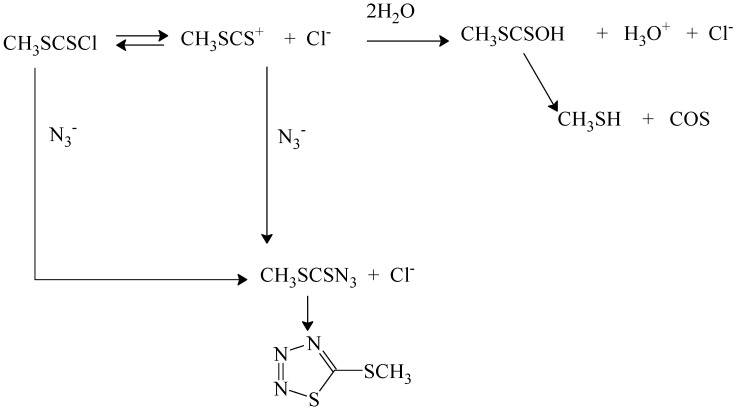
Reactions pathways in 70% acetone containing azide ion for reaction of (methylthio)thiocarbonyl chloride (methyl chlorodithioformate).

The effect of added salts was instructive [82]. Addition of sodium perchlorate gave modest increases in rate, suggesting a positive salt effect. Addition of sodium chloride led to a reduced rate, indicating common-ion return, which must be from free carbocations. Addition of azide ion led to reduced amounts of acid production and formation of products from azide attack (Scheme 5). In this scheme [82,83] the azide product, which spontaneously cyclizes, is produced directly by a bimolecular pathway and indirectly by capturing the carbocation produced. Added chloride ion can compete for capture of the carbocation by an external return pathway or the carbocation can be captured by water from the solvent to give a half-ester, which loses COS and gives the methanethiol. The kinetics in the presence of a relatively large concentration (0.05 to 0.2 mol/L) of azide ion is first-order in substrate and first-order in azide ion [83] and the solvolysis reaction observed in its absence is almost totally suppressed.

Application of the Grunwald-Winstein equation was initially done with fourteen solvents and a good correlation was obtained, with an *l* value of 0.55 ± 0.09, and an *m* value of 0.84 ± 0.08 (*R* = 0.967), for an *l*/*m* ratio of 0.65 [70]. The correlation included runs at 25.0 °C in aqueous TFE as well as in EtOH-H_2_O, MeOH-H_2_O and acetone-water. Subsequently additional values became available and also a value for methan(ol-*d*), allowing a *k*_MeOH_/*k*_MeOD_ ratio of 1.49 to be calculated [84], consistent with the earlier proposed ionization pathway.

**Figure 5:**
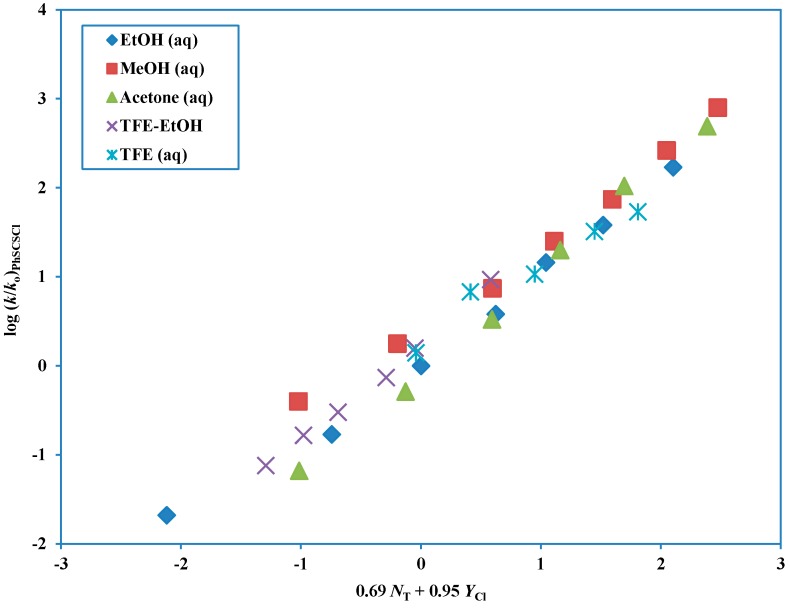
The plot of log (*k*/*k*_o_) for solvolyses of phenyl chlorodithioformate in 31 pure and binary solvents at 25.0 °C against (0.69 *N*_T_ + 0.95 *Y*_Cl_).

With a total of 31 solvolyses now available the correlations were considerably improved and, again using the extended Grunwald-Winstein equation, a good correlation (Figure 5) was obtained for all solvents with an *l* value of 0.69 ± 0.05, *m* value of 0.95 ± 0.03, *c* value of 0.18 ± 0.05, *R*-value of 0.987 and *F*-value of 521 [72] (last entry of Table 3). The *l* and *m* values are both slightly increased relative to the 14 data point correlation, as is the *l*/*m* ratio of 0.72, which remains, however, in the range consistent with an ionization mechanism.

As one would predict, since replacing either oxygen of phenyl chloroformate with sulfur leads to the incursion of an ionization mechanism for solvolyses with fluoroalcohol-rich solvents, replacing *both* oxygens leads to a further increase in the tendency towards ionization, such that it is now observed over the full range of solvents. Accordingly, while PhOCOCl shows an addition-elimination mechanism over the full range of investigated solvents, PhSCSCl follows an ionization pathway over the same range of solvents and the intermediate PhSCOCl and PhOCSCl both show intermediate sovolysis behavior.

In addition to conclusions drawn from the consideration of the ease of ionization, one should also consider ground-state effects. Indeed, the slow reactions of chloroformates relative to acyl chlorides are largely due to ground-state stabilization, through resonance, in the former [1]. Calculations of this stabilization at the HF/6-31G(d) level [79] show a 16 kcal/mol stabilization for methyl chloroformate relative to methyl chlorothioformate, leading to more energy being needed to break the C–Cl bond for a chloroformate, and a faster reaction for the corresponding chlorothioformate in the ionization pathway. The strength of the C–Cl bond will be considerably less important when the addition step of an addition-elimination pathway is rate-determining.

## 5. Carbamoyl and Thiocarbamoyl Halides

Although less obviously related to the other substrates of this review, a brief consideration of the RR'NCOCl and RR'NCSCl compounds, in terms of their behavior under solvolytic conditions, can serve as a check on the explanations given for the effects of replacing oxygen by sulfur.

They can be considered as being related to the ROCOCl and ROCSCl compounds by the replacement of the alkoxy group by an NRR’ amino group or in terms of the replacement of the oxygen of the alkoxy group by an NR' group.

**Scheme 6:**
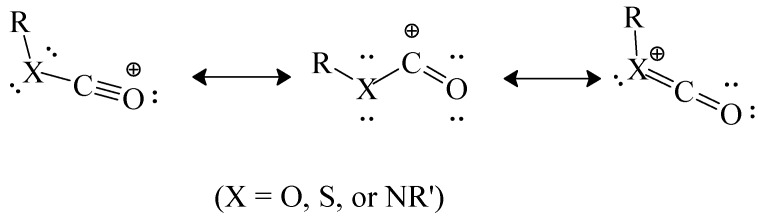
Resonance-stabilized acylium ion.

It has been postulated that the chlorothioformate has a greater tendency to react by ionization than the chloroformate because, in the incipient acylium ion at the transition state, the sulfur can better carry a positive charge within the resonance hybrid that is being formed (Scheme 6). Bentley [54] has carried out calculations which combine these effects of cation stabilization with the ground-state stabilization effects discussed at the end of the immediately preceding section. In this way he arrived at heterolytic bond dissociation energies for a series of phenyl esters. Values (in kcal/mol) were arrived at of 166.5 for PhOCOCl, 153.3 for PhSCOCl, 148.7 for PhOCSCl, and 143.4 for PhSCSCl. These values are nicely consistent with the observed trends of mechanistic change.

One would expect an even greater tendency towards ionization when a portion of the charge is placed on a nitrogen atom, which is even more capable of carrying a positive charge. It is, indeed, found that an analysis of the solvolyses of *N*,*N*-dimethylcarbamoyl chloride [85] gives a good linear correlation in an extended Grunwald-Winstein treatment, with an *l* value of 0.56 ± 0.05 and an *m* value of 0.70 ± 0.04 (*R* = 0.983) for an *l*/*m* ratio of 0.86, consistent with an ionization pathway with a relatively large degree of assistance from nucleophilic solvation, or with a loose S_N_2 transition state, with little bond-making and extensive bond-breaking. Similar behavior was also observed for the *N*,*N*-diphenylcarbamoyl chloride and three *N*-alkyl-*N*-arylcarbamoyl chlorides [72].

A study in terms of the substitution effects in the solvolyses of *N*,*N*-dialkylcarbamoyl chlorides [86] showed that intensifying the electron release from the alkyl group led to faster reactions and the rates could be correlated using the alkyl group σ* values (a scale of the Hammett σ-value type but devised for this type of situation [87]). The ϱ* values obtained were at values of −4.1 in 50% aqueous acetone and of −3.7 in ethanol, consistent with an appreciable assistance to the solvolysis process from an electron-release by the alkyl groups. A series of corresponding thio-derivatives was studied in 70% acetone [88]. The sensitivity to changes in σ* values was again appreciable, but considerably less than in the earlier study [86]. A value for ϱ* of −1.73 was observed.

These differences in magnitude are consistent with a reduced demand for electron density from the amino group when the first of the canonical structures in Scheme 6 (with X = NR') has the oxygen replaced by sulfur which can better carry a positive charge, increasing the contribution of this structure to the overall hybrid. This further increase in stability of the formed carbocation will also reduce the energy content at the transition state and one would expect a faster reaction. This is indeed the case and the ratios of the specific solvolysis rates of (CH_3_)_2_NCSCl relative to those for (CH_3_)_2_NCOCl, at 0.0 °C, of 120 to 344 in ethanol, methanol, and their binary mixtures with water, increasing to 448–1660 in TFE and mixtures of TFE with H_2_O or ethanol demonstrate large effects. In a Grunwald-Winstein treatment [19] using the two-term version (Equation (2)) both the *l* and *m* values are lower for the thiocarbamoyl derivative than for *N*,*N*-dimethylcarbamoyl chloride, with values of 0.29 ± 0.03 for *l* and 0.55 ± 0.06 for *m* (*R* = 0.993). The *l*/*m* ratio of 0.53 is also considerably lower than the value of 0.86 for (CH_3_)_2_NCOCl. These reduced values are consistent with the above discussed more stable cation in the presence of sulfur, leading to a lower energy barrier and an earlier transition state for the ionization process involved in its production.

## 6. Conclusions

The introduction of sulfur in ROCOCl substrates introduces a variety of superimposed mechanisms and the ranges of dominance is dependent on the R group, the presence of one or two sulfurs, and the types of solvent studied (*i.e.*, on the *N*_T_ and *Y*_Cl_ values). On the other hand, *N*,*N*-disubstituted carbamoyl and thiocarbamoyl chlorides favor the ionization pathway.
